# Super-resolution imaging of Douglas fir xylem cell wall nanostructure using SRRF microscopy

**DOI:** 10.1186/s13007-022-00865-3

**Published:** 2022-03-05

**Authors:** Lloyd A. Donaldson

**Affiliations:** Scion, 49 Sala Street, Rotorua, 3010 New Zealand

**Keywords:** Super-resolution radial fluctuations, Cell wall, Lignin, Porosity, Lamellation

## Abstract

**Background:**

The nanostructure of plant cell walls is of significant biological and technological interest, but methods suited to imaging cell walls at the nanoscale while maintaining the natural water-saturated state are limited. Light microscopy allows imaging of wet cell walls but with spatial resolution limited to the micro-scale. Most super-resolution techniques require expensive hardware and/or special stains so are less applicable to some applications such as autofluorescence imaging of plant tissues.

**Results:**

A protocol was developed for super-resolution imaging of xylem cell walls using super-resolution radial fluctuations (SRRF) microscopy combined with confocal fluorescence imaging (CLSM). We compared lignin autofluorescence imaging with acriflavin or rhodamine B staining. The SRRF technique allows imaging of wet or dry tissue with moderate improvement in resolution for autofluorescence and acriflavin staining, and a large improvement for rhodamine B staining, achieving sub 100 nm resolution based on comparison with measurements from electron microscopy. Rhodamine B staining, which represents a convolution of lignin staining and cell wall accessibility, provided remarkable new details of cell wall structural features including both circumferential and radial lamellae demonstrating nanoscale variations in lignification and cell wall porosity within secondary cell walls.

**Conclusions:**

SRRF microscopy can be combined with confocal fluorescence microscopy to provide nanoscale imaging of plant cell walls using conventional stains or autofluorescence in either the wet or dry state.

## Background

Xylem cell walls are characterised by a layered structure of cellulose embedded in a hemicellulose/lignin matrix [1–3]. Typical secondary cell walls in xylem are made up of three layers, an outer S1 layer with a cellulose microfibril orientation approximately perpendicular to the longitudinal axis of the wood fiber, a wide S2 layer with cellulose microfibrils oriented at 10°–30° to the fibre axis, and a narrow, more highly lignified layer lining the lumen of the fibre with cellulose orientation close to horizontal [4, 5]. Outside the secondary wall is a highly lignified layer composed of the primary cell wall and middle lamella, known as the compound middle lamella (CML). The middle lamella is often poorly defined except at cell corners where it is much larger. All xylem cell types including tracheids, fibres, vessels, and some parenchyma cells follow this common pattern but with modifications in reaction wood where the composition and layering may be different [3, 6–9]. Bast fibres from dicotyledonous phloem or bark such as hemp or linen also have 3-layered secondary cell walls with a thick S2 layer [10].

Bamboo parenchyma, phloem, and xylem fibres have polylamellate cell walls with multiple layers associated with changes in microfibril orientation, lignification, and amount of xylan [2, 11–15]. Polylamellate cell walls associated with changes in microfibril angle are known as helicoidal cell walls because of the continuous rotation of microfibril orientation relative to the cell axis. This arrangement occurs notably in the stone cells of pear fruit where up to 100 layers have been reported [16].

Nanolamellae, either circumferential or radial, have been described in tracheid and fibre cell walls using atomic force microscopy (AFM) but these are too small to be detected by light microscopy and reflect the organisation of nanoscale structures of cellulose and lignin [17–19]. Observations on lamellation and microfibril orientation by AFM have been performed under water-immersion conditions but mainly on chemically modified (delignified) cell walls [20–22].

Cell walls undergo swelling and shrinkage in response to changes in water content and may also undergo irreversible structural changes as a result of drying [23–26]. The ability to image the nanostructure of wood cell walls in the never-dried native state is therefore of considerable interest [27]. While it is possible to examine wet cell walls in an environmental or cryo scanning electron microscope (SEM), the water film is opaque to electrons and hence obscures ultrastructural details [28]. Light microscopy enables imaging of wet cell walls but with limited resolution [29].

Super-resolution microscopy achieves sub-diffraction limit resolution often in the tens of nanometers providing images close to the detail resolved by electron microscopy but with often simple sample preparation, and imaging under more natural conditions is possible potentially allowing live-cell imaging [29–31]. There are a range of implementations most of which require specialised hardware and/or specific types of fluorescent probes which can limit potential applications. Super-resolution microscopy has been applied to plant cell biology to image the cytoskeleton and organelles such as endoplasmic reticulum, mitochondria, and plastids in living cells [32]. Plant cells present some challenges to the application of super-resolution techniques, including refractive index mismatches between cell walls (1.53–1.59) and mounting medium (Glycerol = 1.47) which contribute to spherical aberration and light scattering [30, 32, 33].

Only one study has previously applied super-resolution microscopy to plant cell walls. In poplar xylem, stimulated emission depletion (STED) and deconvolution were used to investigate cell wall layers [34]. This allowed visualisation of secondary walls and middle lamella with resolution intermediate between light and electron microscopy.

Super-resolution radial fluctuations (SRRF) avoids the limitations of other techniques such as the requirement for specific hardware and specialised fluorescent probes. SRRF analysis can be applied to either confocal or widefield fluorescence microscopy and potentially any fluorescent probe [35, 36]. SRRF analysis is primarily an image processing method but can also benefit from the use of sCMOS (scientific Complementary Metal–Oxide–Semiconductor) cameras for faster real-time live imaging using the SRRF-stream application developed by Andor Technologies.

SRRF analysis has had limited application to plant cell biology. Galvan-Ampudia et al. [37] used SRRF microscopy to study cell polarity in flower primordia of *Arabidopsis* using a GFP labeled PIN1, an auxin efflux carrier protein. Huokko et al. [38] used SRRF-stream microscopy to examine the dynamics of thylakoid membranes in cyanobacteria.

In the current investigation, we applied SRRF microscopy to nanoscale imaging of secondary xylem cell walls in the wet state and compared autofluorescence with acriflavin and rhodamine B staining.

## Results

### Resolution

The objective lens resolution (full width at half maximum height, FWHM) for the 63×/1.4 NA oil immersion lens was measured as 235 nm in x, y, and 567 nm in z. The theoretical resolution (Abbe) of this lens is 160 nm at 561 nm excitation wavelength in x, y, and 401 nm in z. For comparison, the theoretical resolution of the 63×/1.45 NA glycerol immersion lens used for wet cell wall imaging at 561 nm excitation wavelength is 155 nm in x, y, and 374 nm in z, and for the 63×/1.20 NA water immersion lens is 187 nm in x, y, and 545 nm in z.

**Fig. 1:**
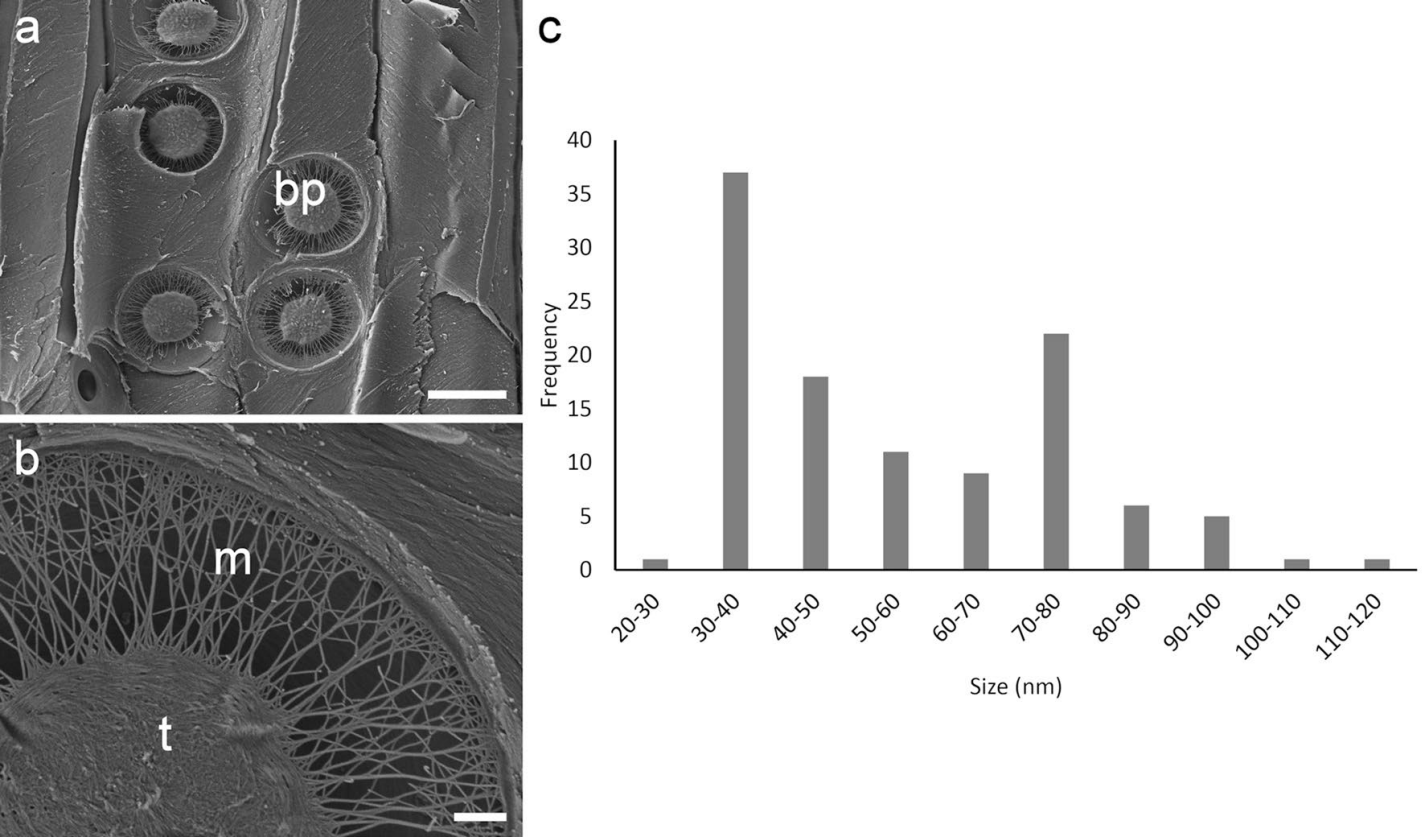
Measurement of margo fibrils by SEM indicates an average thickness of 56 nm and a range of 20–120 nm. **a** Bordered pits (bp) in radial longitudinal view. Scale bar: 10 μm. **b** A close-up view of the torus (t) and margo (m). Scale bar: 1 μm. **c** Frequency distribution of margo fibril diameter

**Fig. 2:**
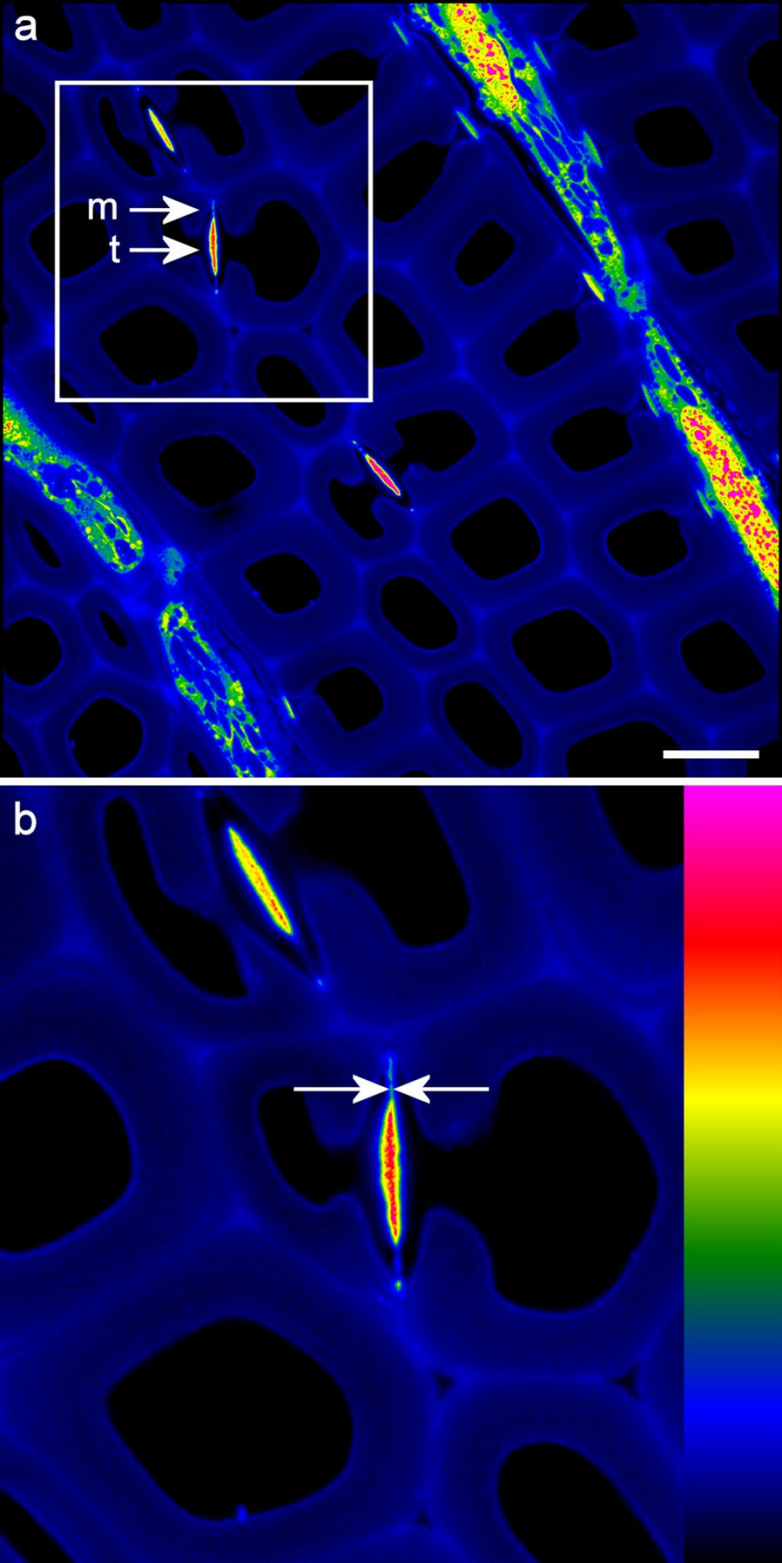
Measurement of margo fibrils by autofluorescence SRRF indicates a thickness range of 70–120 nm. **a** Pit membranes in transverse view showing margo (m) and torus (t). **b** Enlarged view of **a**. The distance between the arrows is 70 nm. Scale bar: 10 μm. Intensity scale: 0–255

Measurement of bordered pit margo fibrils by SEM indicated a range of diameter from 20 to 120 nm with an average of 56 nm (Fig. 1). The size distribution showed two frequency peaks at 30–40 nm and 70–80 nm. Measurements of margo fibrils by SRRF microscopy using the 63 × 1.4 NA oil immersion lens indicated a thickness of 70–120 nm (Fig. 2) suggesting that SRRF analysis improves x, y resolution from 235 to < 100 nm. Margo fibrils were measured in the radial view by SEM and in transverse view by fluorescence microscopy due to sample preparation considerations. Because these fibrils are round in shape and form more or less in a monolayer these measurements are considered to be comparable.

### SRRF algorithm

**Fig. 3:**
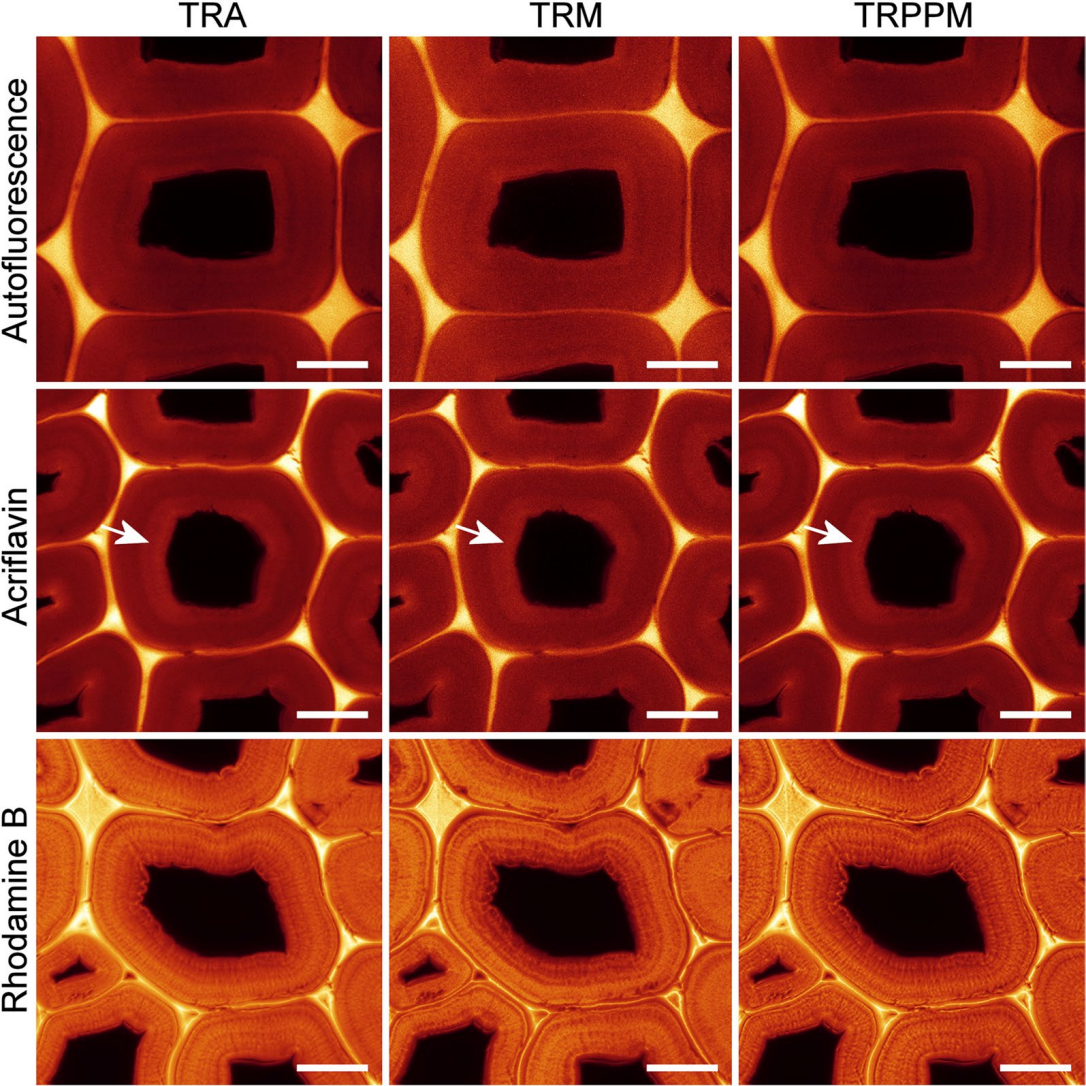
Comparison of SRRF algorithms as applied to autofluorescence, acriflavin, and rhodamine B stained sections. The TRPPM algorithm combined with rhodamine B staining gives the best results in terms of image detail and the absence of artefacts. Autofluorescence, acriflavin, and rhodamine B staining all detect slight variations in lignification within the S2 layer (arrows). Scale bars: 10 μm

The SRRF software offers a choice of four different algorithms. Initial comparisons indicated that the temporal radiality autocorrelation (TRAC) algorithm gave unrealistic grainy images. A more detailed comparison of the other three options also indicated grainy results for temporal radiality maximum (TRM). Good results were obtained with temporal radiality average (TRA) and temporal radiality pairwise product mean (TRPPM) with sharper details in TRPPM images indicating an optimum result (Fig. 3). Therefore, the TRPPM algorithm was used for all subsequent SRRF analysis. Varying the parameters in the software from the default values did not yield any significant improvement.

### Staining

**Fig. 4:**
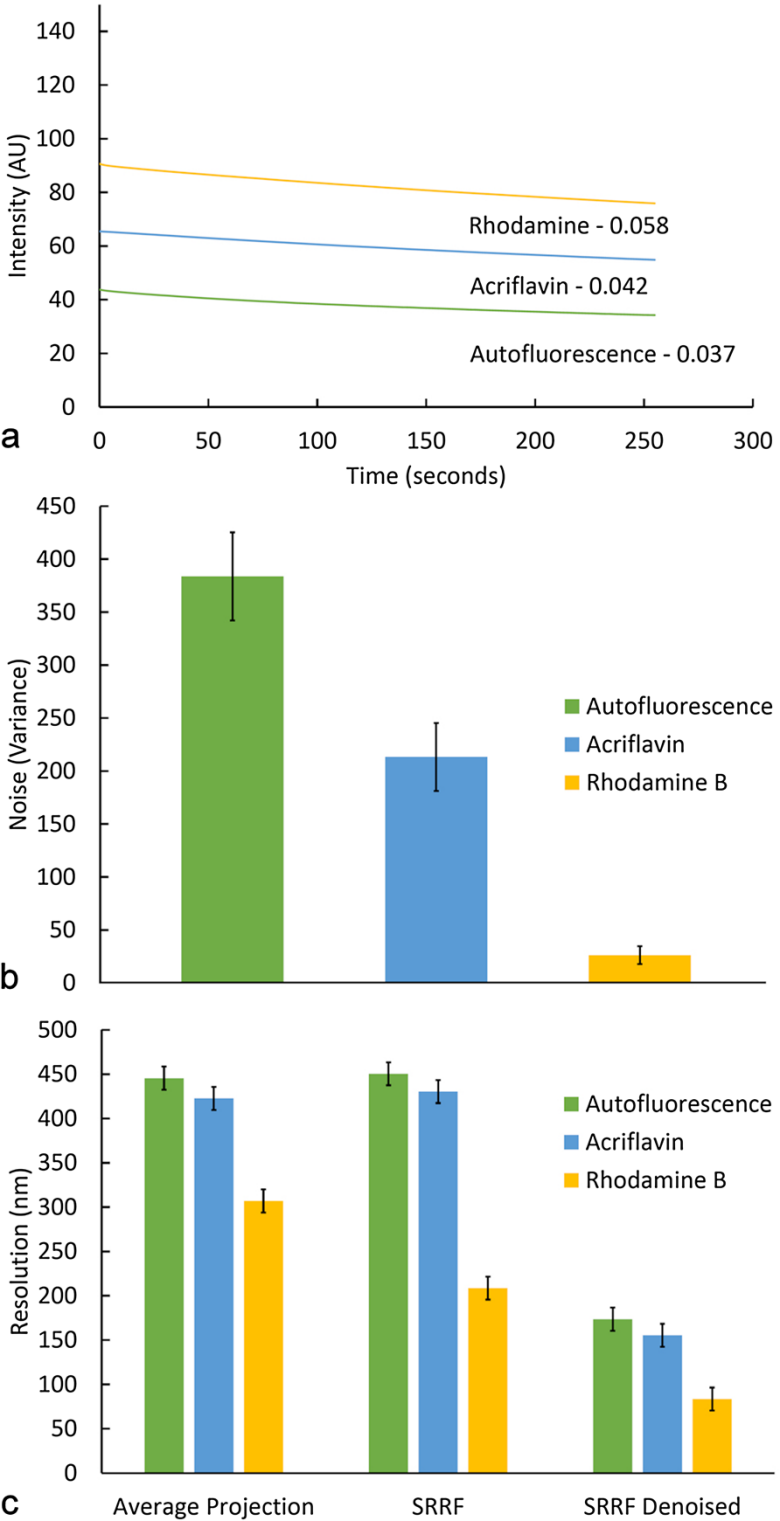
**a** A comparison of photobleaching during the 255-s exposure for SRRF imaging. Bleaching rate (grey-levels/second) is comparable for autofluorescence, acriflavin, and rhodamine B. **b** Noise levels as measured by pixel variance in a representative region of interest of cell wall from a single confocal image with no averaging. Autofluorescence and acriflavin images show significantly more noise compared to rhodamine B. **c** Resolution determined by FRC analysis comparing CLSM (average projection) with SRRF and SRRF + Denoising

A significant advantage of SRRF analysis when applied to plants is the ability to use autofluorescence for label-free imaging. Lignin autofluorescence allowed imaging of wall layers and detection of slight changes in lignin concentration across the secondary cell wall in the SRRF images (Fig. 3). SRRF processing of autofluorescence also allowed improved resolution of margo fibrils in bordered pit membranes due to their very bright and stable autofluorescence probably arising from extractives (Fig. 2) [39]. Individual confocal images of lignin autofluorescence had the greatest amount of noise as measured by between pixel variance (Fig. 4) and this resulted in less apparent improvement in resolution by SRRF processing compared to stained samples.

SRRF analysis of acriflavin staining yielded a similar improvement to lignin autofluorescence but with less noise in the individual confocal images (Fig. 4). Acriflavin staining allowed improved resolution of cell wall layers and detection of small changes in secondary wall lignification across the cell wall (Fig. 3). No novel structures were revealed in SRRF images from autofluorescence or acriflavin staining.

**Fig. 5:**
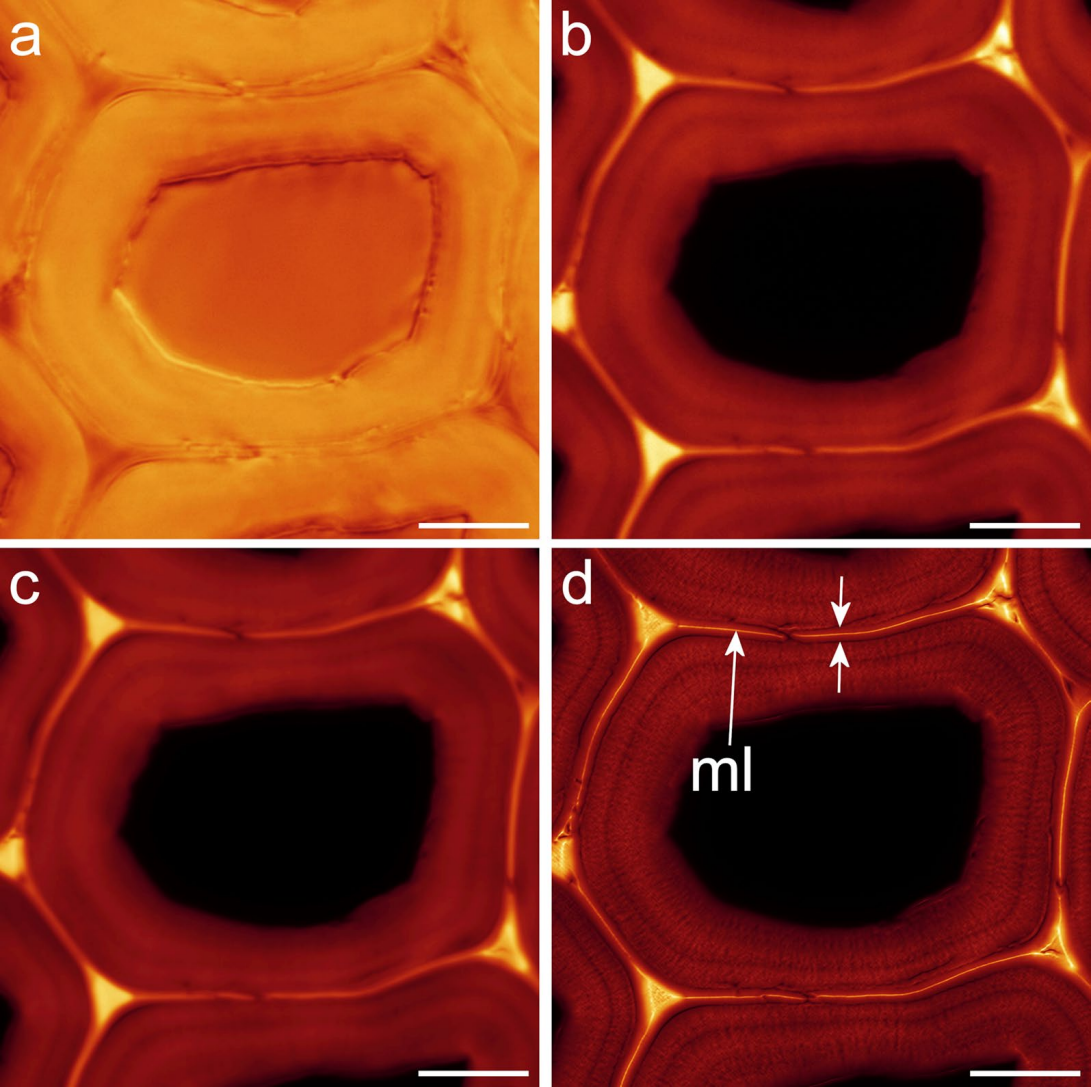
**a** Brightfield transmission image of rhodamine B stained latewood. **b** Single confocal slice image. **c** Average intensity projection of 100 confocal slices. **d** SRRF image. The SRRF image differentiates the middle lamella (ml) and a wide layer between the two short arrows that may include both the S1 layer and primary cell wall, 1–1.5 μm wide. Scale bars: 10 μm

Rhodamine B staining produced bright, noise-free confocal images with minimal photobleaching (Figs. 3, 4). A comparison of brightfield transmission, confocal fluorescence projection, and SRRF images of the same field of view for a rhodamine B stained section is shown in Fig. 5 and demonstrates the increase in detail visible after SRRF processing. SRRF analysis resulted in a large improvement in resolution revealing enhanced details of lignin distribution as well as concentric and radial lamellae within the secondary cell wall that were not detected by autofluorescence or acriflavin (Fig. 3). This resulted in new information on the nanostructure of wet cell walls related to the clustering and arrangement of cellulose fibrils as previously described by electron microscopy and AFM [17, 28]. SRRF imaging of rhodamine B stained cell walls, therefore, provides information on the nanostructure of wet cell walls comparable to that from electron microscopy studies which are generally restricted to dry cell walls. This technique confirmed the presence of radial lamellae within native cell walls not subjected to fracturing, or chemical modification.

Fourier ring correlation (FRC) analysis confirmed the limiting effect of noise in autofluorescence and acriflavin images (Fig. 4c). This method estimates image resolution by comparing two replicate SRRF images of the same field of view acquired under identical conditions and indicated that SRRF processing does not increase the average image resolution for autofluorescence or acriflavin images but did increase the resolution for rhodamine B. Comparison of average projections indicated that rhodamine images have higher resolution compared to autofluorescence and acriflavin even without SRRF processing. Combining SRRF with denoising resulted in significant improvement in resolution for all three techniques indicating that image noise is a limiting factor in this analysis (Fig. 4c). However, SRRF images generated from sequences subjected to PureDenoise contained artefacts in the form of line patterns so this is not an ideal solution. Line averaging, reduced scan speed or the use of high quantum efficiency detectors offer alternative solutions to this problem. There may also be other alternative staining methods that could yield results comparable to rhodamine B.

**Fig. 6:**
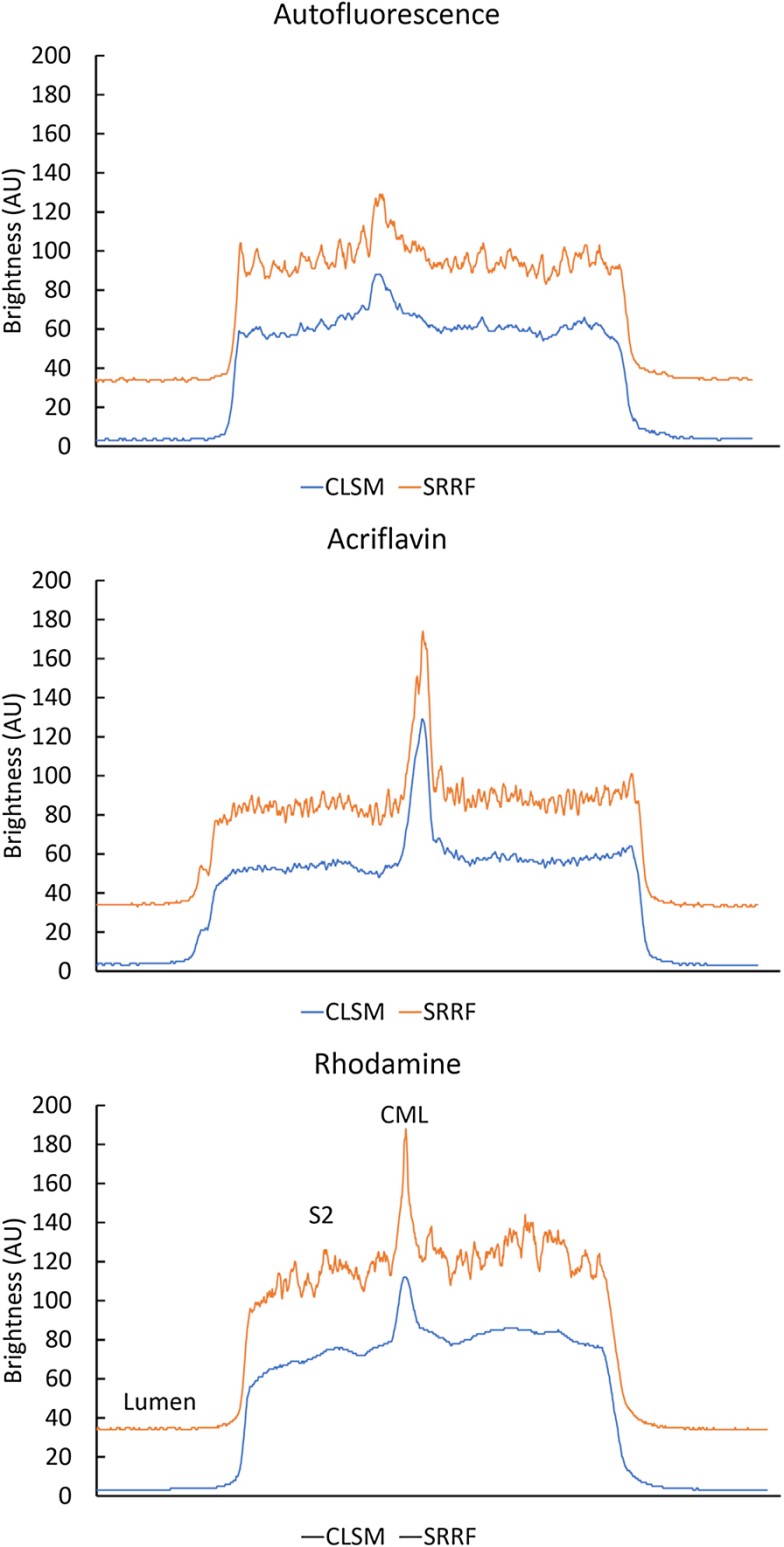
Brightness profile comparison between a confocal average projection and the corresponding SRRF image across a cell wall at a matched location. Autofluorescence and acriflavin staining show a slight increase in signal/noise while rhodamine shows a large increase in detail

Line profiles across cell walls from lumen to lumen at matched locations on average projections and SRRF images demonstrate both the enhanced image detail in SRRF images as well as showing differences in signal to noise between staining methods with relatively low contrast in autofluorescence compared to acriflavin and rhodamine B (Fig. 6). This analysis confirms that detail is increased only in the cell wall region with no change in the empty lumen so this effect is unlikely to represent noise enhancement that would occur in both parts of the image.

### Artefacts

SRRF images were sensitive to overexposed regions for example in the middle lamella where some distortions/patterns were occasionally observed so it is important to carefully adjust brightness before acquisition while still allowing for expected photobleaching during the 4-min time series exposure.

### Measurements

One application for SRRF imaging is to improve the accuracy of measurements of cell wall dimensions, potentially to assess the thickness of different cell wall layers or pit membranes under wet conditions without the use of electron microscopy. We compared measurements of the compound middle lamella (middle lamella + primary cell walls) using autofluorescence, acriflavin, or rhodamine B by average projection and SRRF, using measurements with SEM as a reference. Measurements showed a dependence of variance on the mean and hence were log-transformed.

Average projection and SRRF measurements were location matched and SRRF measurements were consistently smaller than confocal measurements (Table 1). Some differences were observed between the three imaging methods. Acriflavin staining tended to give a higher estimate of CML width compared to autofluorescence in average projections possibly because differentiation of S1 and CML layers was less clear but SRRF processing seemed to correct this problem. Rhodamine B staining conversely gave significantly smaller measurements after SRRF processing whereas measurements on average projections were comparable to autofluorescence and acriflavin staining. These differences probably reflect differences in the exact structures being highlighted by each method. In the case of rhodamine B staining, it is unclear exactly what this narrow layer represents since a similar-sized structure could not be detected by SEM imaging of the same samples (Fig. 5d). Measurements from autofluorescence and acriflavin staining performed on SRRF images of dry cell walls were similar to measurements made by SEM whereas measurements on average projections were significantly larger. All three methods were capable of detecting shrinkage of the CML as a result of drying.

**Table 1:** Measurements of cell wall layers

	Wet			Dry		
	Autofluorescence	Acriflavin	Rhodamine B	Autofluorescence	Acriflavin	Rhodamine B
SRRF	408	552	144	456	384	144
	432	624	144	288	480	144
	384	480	120	360	288	144
	408	480	120	360	288	120
	360	456	144	360	288	144
	624	456	264	432	312	120
	480	624	240	336	336	120
	360	360	240	384	288	144
	648	384	120	480	384	120
	576	456	168	336	360	120
	**468**^**a**^	**487**^**a**^	**170**^**d**^	**379**^**e**^	**341**^**f**^	**132**^**g**^
CLSM	480	936	528	552	600	408
	624	744	576	408	864	480
	456	624	600	432	600	384
	504	720	432	432	840	432
	648	600	648	432	552	432
	744	672	864	504	576	384
	624	840	888	456	552	480
	480	480	912	456	552	456
	696	504	408	528	576	432
	648	624	552	504	600	384
	**590**^**b**^	**674**^**c**^	**641**^**c**^	**470**^**a**^	**631**^**c**^	**427**^**a**^
SEM	337					
	300					
	337					
	375					
	337					
	337					
	347					
	319					
	356					
	291					
	**334**^**f**^					

Measurements of the width of the compound middle lamella using a paired comparison of SRRF and CLSM (average projection) at matched locations for wet and dry conditions compared to reference measurements by SEM. Methods were significantly different as were interactions between image type and method, p << 0.001. Means with different letters are significantly different for p = 0.05

### Lamellae

**Fig. 7:**
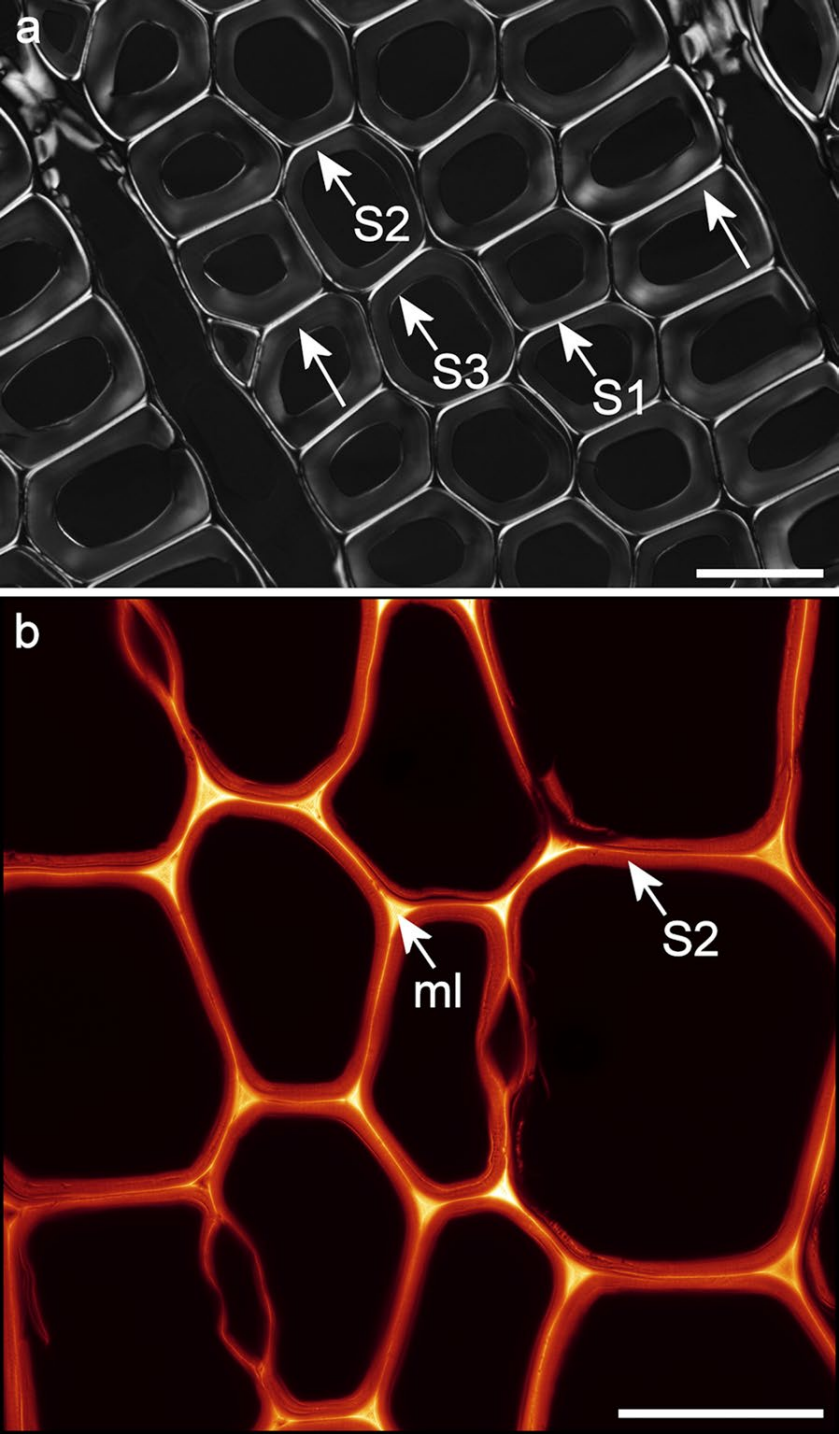
**a** Polarised light image showing cell wall layers. Long arrows show lamellae within the S2 layer. Scale bar: 30 μm. **b** Rhodamine B stained SRRF image of earlywood showing middle lamella (ml) and S2 layer. Scale bar: 30 μm

Concentric lamellae within the S2 layer show reduced birefringence when viewed by polarised light microscopy indicating that these structures contain either less cellulose (and hence more lignin) or cellulose at slightly different orientation and are unrelated to S1 and S3 layers (Fig. 7). Their darker appearance with rhodamine B staining suggests that these are regions of altered lignin or porosity that might be associated with the margins of slight variations in lignification observed in corresponding images using autofluorescence and acriflavin staining.

**Fig. 8:**
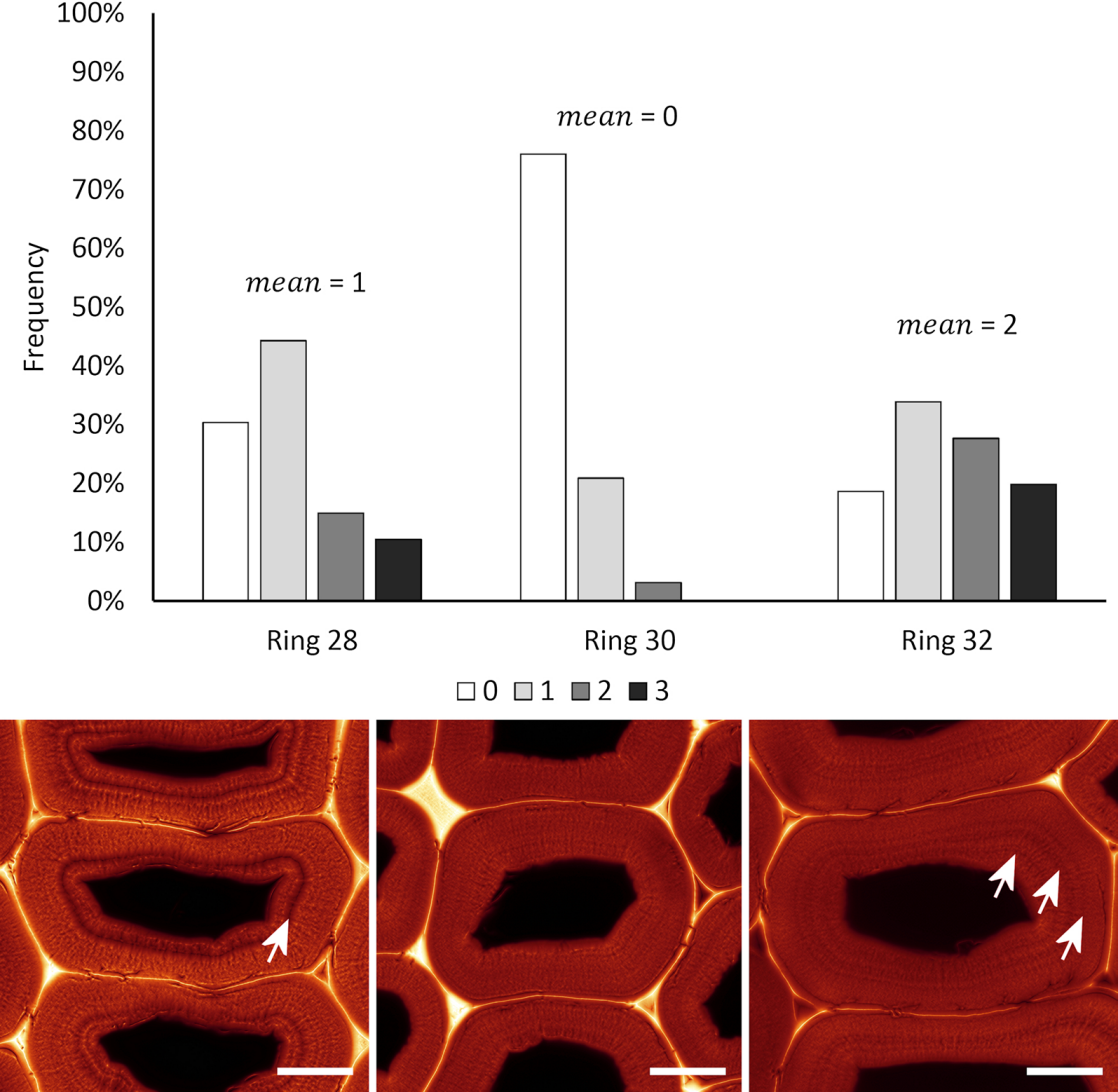
Analysis of concentric lamella patterns in growth rings 28, 30, and 32 based on samples of 200, 225, and 242 cells respectively. In growth ring 28 a single prominent lamella near the lumen is common (arrow), in growth ring 30 there are only indistinct lamellae, whereas in ring 32 there are often 2 or 3 moderately distinct lamellae (arrows). Patterns are generally similar among adjacent cells. Variation in cell wall structure between growth seasons suggests that these structures may be related to the environment. Scale bars: 10 μm

Comparison of three adjacent growth rings revealed a tendency for different patterns of concentric lamellae as revealed by SRRF analysis (Fig. 8). Tracheid secondary walls in latewood from ring 28 had up to three concentric lamellae, two closer to the lumen and one closer to the periphery or in some cases equally spaced. On average there was one lamella per cell. Secondary walls in ring 30 generally had indistinct concentric lamellae but occasionally there were one or two distinct lamellae near the centre of the wall or towards the lumen. Secondary walls in ring 32 had up to three distinct lamellae with on average two lamellae per cell. There was a tendency for consistent patterns among adjacent cells in radial files. Small cells representing tracheid tips typically lacked distinct lamellae as did thin earlywood cell walls (Fig. 7).

**Fig. 9:**
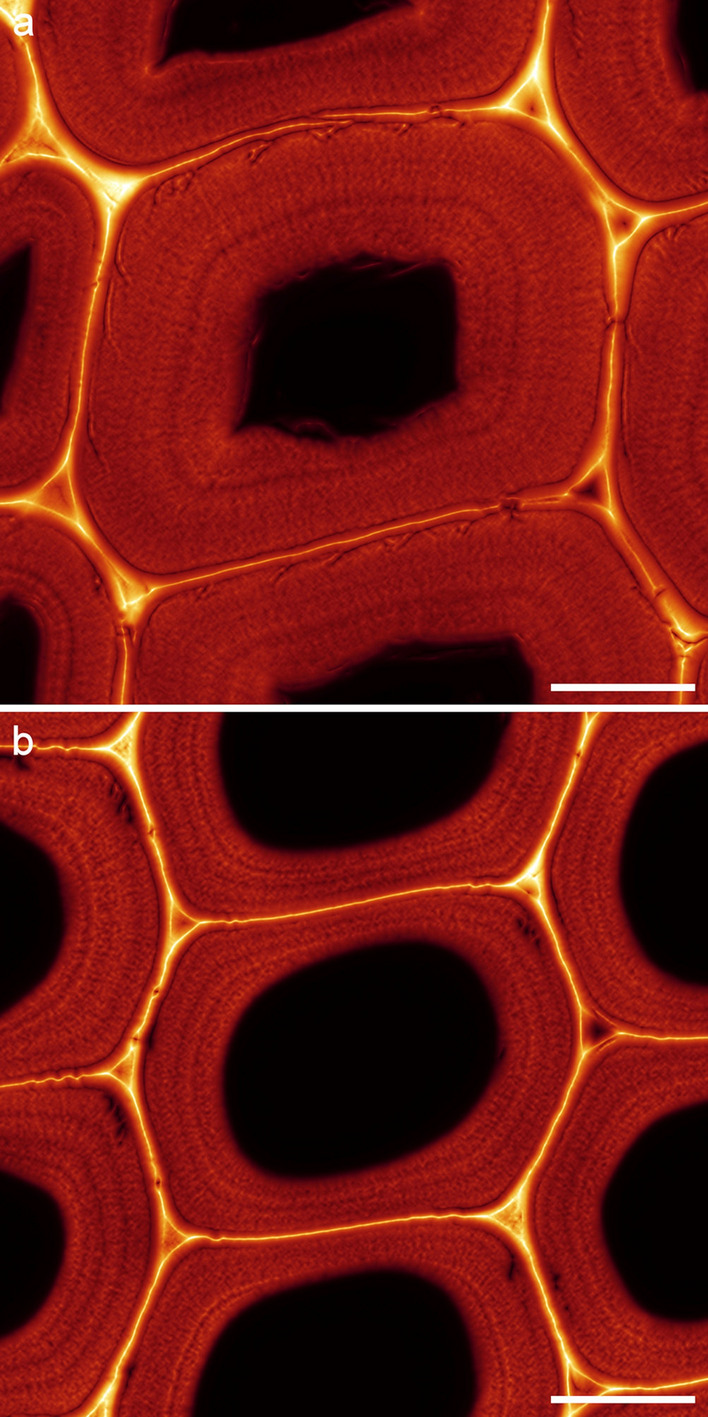
**a** A SRRF image of rhodamine B stained latewood mounted in water. Scale bar: 10 μm. **b** SRRF image of rhodamine B stained latewood after ethanol drying mounted in immersion oil. Scale bar: 10 μm

Comparison of SRRF images from rhodamine B stained sections in glycerol or water confirmed that lamellae were not the result of any swelling that might be induced by mounting in glycerol (Fig. 9). Imaging of carefully dried sections mounted in immersion oil demonstrated that lamellae were consistently present and did not seem to change as a result of drying (Fig. 9).

## Discussion

Imaging of autofluorescent nanofibrils in pit membranes indicated improved resolution after SRRF analysis. Structures close to 70 nm could be detected which is similar to resolution values reported in other studies using fixed cells [36]. Comparison of margo fibril diameter measurements by SEM and by SRRF analysis confirmed that SRRF analysis could distinguish the larger fibrils in the size distribution between 70 and 120 nm. It was easier to image these structures in cross-section (after embedding) than on radial surfaces due to the presence of autofluorescent pit borders in front of and behind the pit membrane which greatly reduced contrast in the radial view. Pit membranes could be detected on radial surfaces after rhodamine B staining thus potentially allowing a wet/dry comparison.

Comparison of different SRRF algorithms indicated that TRPPM gave the best image quality being sharper than TRA and less grainy than TRM. The default parameters were used as changing these either offered no improvement or reduced perceived image quality. Drift correction was unnecessary as sections were stable and did not move during image acquisition. Using fewer than 50 images in the temporal sequence resulted in a slight loss of image quality. Using a reduced number of images might be a slight advantage in cases of rapid photobleaching but using more than 100 images did not yield any obvious benefit. Using fewer pixels in the original temporal sequence reduced the effectiveness of the SRRF processing as expected. Not surprisingly, optimising the resolution of the original data maximised the result of SRRF processing. FRC analysis demonstrated the importance of noise on the resolution attainable by SRRF with the relatively noisy autofluorescence and acriflavin signals having less resolution than low noise rhodamine B images.

We compared SRRF imaging of lignin autofluorescence with acriflavin staining. These two methods both detect lignin but have different signal/noise, with autofluorescence having less contrast between the secondary wall and middle lamella compared to acriflavin staining. This difference probably accounts for the overestimation of CML thickness by acriflavin in average projections compared to autofluorescence since the measurements are dependent on intensity differences.

Autofluorescence and acriflavin staining could detect the S3 layer when present, however, the S1 layer was difficult to resolve. To distinguish the CML and ensure that the structure observed in SRRF images did not include the S1 layers which could look similar, measurements were made on polarised light images where the S1 layer was easily resolved. The width of the double S1 layers and CML was about 1.5 μm confirming that SRRF measurements of 4–500 nm did not include the S1 layers and thus accurately represent the compound middle lamella. However, for rhodamine SRRF images the central part of the cell wall was resolved into at least two structures, a very thin layer 140 nm wide probably at least part of the CML that was more reactive to the rhodamine B dye, and a much wider layer on either side of the middle lamella, the total structure 1000–1500 nm wide likely includes the S1 layers (Fig. 5). Correlative microscopy comparing SRRF and transmission electron microscopy (TEM) images could help elucidate exactly which layers are detected.

Rhodamine B staining is known to adsorb to both lignin and mannan but is somewhat limited by the accessibility of cell walls to the disc-shaped rhodamine molecule, so this signal not only reflects the degree of lignification but is also related to porosity [40, 41]. For example, rhodamine B stained samples often show dark cell corners which represent the limited accessibility of the highly lignified middle lamella to the stain [41]. Rhodamine B staining detected nano-features including concentric and radial lamellae not clearly visible in autofluorescence or acriflavin images. Rhodamine B staining also gave a significantly lower value for CML thickness in SRRF images (but not in average projections) and also clearly detected the S1 layer which is indistinct in autofluorescence and acriflavin images. Rhodamine B staining, therefore, provides more highly detailed images of nanostructure compared to autofluorescence and acriflavin which are better at visualising slight changes in lignification across the secondary cell wall.

Measurement of cell wall layers by SRRF analysis agreed with SEM measurements of the CML while comparative measurements between wet and dry states were able to detect the small amount of shrinkage present in this highly lignified part of the cell wall. Using the FWHM method for measurements of wall layer thickness we found that measurements were slightly overestimated compared to direct visual assessment using a measurement cursor (as used for SEM measurements). While this method avoids observer bias it is probably not ideal for this type of measurement as some layers are distinguished by texture rather than intensity. SRRF analysis should allow accurate measurement of cell wall layers to the nearest 100 nm. As applied to digital images, Nyquist sampling requires a pixel size of at least one-third of the size of the smallest resolved object [29]. A pixel size of 24 nm provides sufficient Nyquist sampling for such measurements, but it is feasible to improve pixel size to 10 nm for even greater precision by reducing the field of view.

Only three different fluorophores were compared in the current study. However, SRRF analysis is likely to work with almost any stain subject to noise and fading behaviour including cell wall stains such as calcofluor, Congo red (cellulose stains), and safranine (bichromatic stain for lignin and cellulose) [42–44]. SRRF processing could also be applied to the immunolocalization of cell wall molecules [41].

The only previous application of super-resolution microscopy to cell walls utilised stimulated emission depletion (STED) microscopy [34]. The authors found that rhodamine-labeled polyethylene glycol could be localised in poplar cell walls yielding images with a similar appearance to our rhodamine SRRF images. STED microscopy is restricted to dyes that specifically respond to the depletion laser and hence this technique is unlikely to work at all with autofluorescence [39].

Some of the concentric lamellae observed with SRRF analysis of rhodamine B staining were associated with the boundaries between lignification levels within the S2 layer. Similar variations in lignification within the secondary cell wall have been reported in bamboo fibres by TEM [2, 11–15]. However, concentric lamellae in Douglas fir latewood cell walls are indistinct by conventional light microscopy and have not previously been described. Under polarised light microscopy, concentric lamellae do not show birefringence suggesting they are not associated with cellulose orientation. These structures may represent slight changes in cell wall porosity or lignification.

Some plant cell walls are known to contain concentric lamellae at different scales [14, 16, 17]. Observations by TEM often show lamellae consisting of individual cellulose microfibrils and matrix materials on a scale of about 3 nm [1, 18]. Methods such as SEM and atomic force microscopy (AFM) detect larger lamellae on cut or fractured surfaces associated with bundles of cellulose microfibrils known as macrofibrils which are typically 30 nm or greater in size [17, 28, 45]. Radial lamellae associated with lignin structures have also been observed by TEM [18], and by SEM on fractured surfaces [46]. There is some debate about whether the radial patterns on fracture surfaces are induced by the fracturing process or whether they are present also in the native cell wall. Our observations using rhodamine SRRF suggest that weak radial lamellae occur in native cell walls using confocal imaging focussed several microns below the cut surface and hence avoiding surface artefacts that might be erroneously detected by AFM or SEM.

We found that adjacent cells within growth rings show a tendency to display similar patterns of concentric lamellae while displaying different patterns in adjacent growth rings. This suggests that these features may be related to environmental conditions. While methods to quantify these patterns need to be further developed our observations suggest the possibility that concentric lamellae are potential climate markers. Since latewood is formed in late summer the possibility of relationships to drought should be investigated. In pine grown under conditions of severe drought stress, tracheids showed abnormal lignification including concentric lamellae within the secondary cell wall very similar to the structures observed in Douglas fir latewood [47]. Because of the inherently layered structure of cellulose in secondary cell walls, lamellae may occur either naturally or as a result of chemical/mechanical treatments such as pulping or by wood decay [17, 48, 49]. Cell wall lamellae can be readily observed by conventional light microscopy as well as by electron microscopy and scanning probe microscopy. Cell wall lamellae therefore have a wide range of origins and have been commonly described in both softwood tracheids and hardwood fibres.

## Conclusions

SRRF analysis allows super-resolution imaging of plant cell wall layers and lamellar structures within the secondary cell wall of xylem. This technique permits more accurate measurement of cell wall layers with the potential to measure shrinkage and swelling behaviour by comparing images acquired in the wet or dry state. SRRF processing can be used in conjunction with autofluorescence as well as conventional staining and therefore could have wide applications in plant structural studies.

## Materials and methods

### Resolution testing

The spatial resolution (Abbe) of a Leica Plan Apochromat 63×/1.4 NA oil immersion objective lens was measured using 90 nm fluorescent silica beads (Sigma 797944) to determine the point spread function. A suspension of beads in distilled water was dried onto a microscope slide made from low-fluorescence glass (clear white glass—Knittel Glass, Germany). Beads were mounted in immersion oil containing antifade (Citifluor AF87) using a #1.5 coverglass (Knittel Glass, Germany). The excitation wavelength was 561 nm and the emission range was 600–800 nm. Images were acquired with a pixel resolution of 48 × 48 × 84 nm in x, y, z at 12-bit dynamic range. Resolution (FWHM) in the x, y, and z planes was determined using the MetroloJ plugin for Fiji software [50, 51]. The theoretical resolutions (Abbe) for three different objective lenses (Leica 63× glycerol, water, and immersion oil lenses) were also calculated using the same software with an excitation wavelength of 561 nm and the numerical aperture value for each lens.

To assess the spatial resolution of xylem cell wall images processed by SRRF analysis, a small branch from a mature Douglas fir tree growing in Rotorua, New Zealand, was fixed in glutaraldehyde for 1 h at room temperature, dehydrated in ethanol, and embedded in LR White resin. The resin block was then polished to produce a microscopically flat transverse surface of the whole stem including xylem, phloem, and bark [52]. The surface of the resin block was mounted in immersion oil with a #1.5 coverslip for confocal imaging of autofluorescent pit membranes. Confocal images were subsequently processed by SRRF analysis and the thickness of the margo fibrils in the pit membrane was measured using Digital Optics V++ software (www.digitaloptics.co.nz) (FWHM). The reason for performing the fluorescence and SEM measurements on the freshly collected branch rather than doing this on the main samples was that the main samples had been previously dried and hence pit membranes were aspirated (closed) making it almost impossible to see the margo fibrils by either method. Measurements were performed on normal wood, the compression wood on the lower side of the branch was avoided.

Samples from the same stem were dehydrated in ethanol, transferred to t-butyl alcohol, and vacuum dried to preserve unaspirated pit membranes [53]. Fractured radial surfaces were prepared for SEM by coating with 5 nm of chromium using a Cressington 208 HR sputter coater equipped with a quartz crystal film-thickness monitor. Bordered pit membranes were examined at 3 kV and 12 kx (8 nm pixel) magnification to measure the diameter of margo fibrils using a JEOL 6700 field emission scanning electron microscope. SEM images were measured using Digital Optics V++ software (FWHM).

### Xylem samples for SRRF analysis

Microscopic analyses were carried out on a single tree of Douglas-fir (*Pseudotsuga menziesii* var. *menziesii/viridis*) grown in the Southwest of Germany from a disc sampled at breast height. The site was part of a spacing trial with tree densities varying between 500 and 4000 trees per hectare [54]. Air-dried samples from three sapwood growth rings, ring numbers 28, 30, and 32 from the pith were re-saturated in water and subsequently stored in 70% ethanol. Blocks washed briefly in water were sectioned for microscopy in the transverse plane at 25 µm thickness using a sledge microtome. The main focus was on latewood because the thick cell walls facilitate intra-wall characterization. Compression wood was completely absent from these samples.

Sections for lignin autofluorescence were mounted in 50% glycerol in 0.01 M phosphate buffer at pH9 [55]. Sections for lignin staining were treated with 2.7 μM (aq.) acriflavin for 10 min followed by washing in water and were mounted in 50% glycerol in 0.01 M phosphate buffer at pH7. Sections for lignin/porosity staining were treated with 1.6 μM (aq.) rhodamine B for 18 h followed by washing in water and were mounted in 50% glycerol in 0.01 M phosphate buffer at pH7. Some comparisons were made by mounting in water, or immersion oil (Citifluor AF87) following ethanol dehydration and air-drying. Sections mounted in glycerol were examined with a Leica SP5 confocal microscope using a Plan Apochromat 63×/1.45 NA glycerol immersion objective lens at 2 × zoom with a pixel size of 120 nm. For sections mounted in water, a 63×/1.2 NA water immersion lens was used whereas for sections mounted in immersion oil a Plan Apochromat 63×/1.4 NA oil immersion lens was used. Ten fields of view were acquired at 1024 × 1024 pixels as a time series of 100 frames at a single focal plane close to the surface of the section. The acquisition time for each field of view was 4 min and 15 s. For acriflavin, the excitation was 458 nm and the emission range was 500–700 nm whereas for lignin autofluorescence the excitation was 488 nm and the emission range was 500–650 nm. For rhodamine B the excitation was 561 nm and the emission range was 600–800 nm. The confocal pinhole size was set to the optimum value of 1 Airy unit, scan speed was 400 Hz, and no line averaging was used.

An average intensity projection was created from each time series to serve as a control image to compare with the SRRF image. The confocal time series were then processed using Fiji software with NanoJ SRRF v 1.14 [56] on a personal computer equipped with an Nvidia RTX 2060 graphics card allowing OpenCL acceleration of the image processing (15 s, compared to ~ 20 min without acceleration). Preliminary testing confirmed that three of the four available algorithms (temporal radiality average, TRA; temporal radiality maximum, TRM; temporal radiality pairwise product mean, TRPPM) gave acceptable results. These algorithms were then applied to the 10 different time series for each of the fluorophores (autofluorescence, acriflavin, and rhodamine B) using the default parameters (ring radius 0.50, radiality magnification 5×, axes in ring 6) except that radiality renormalisation was active. Drift correction was not required. The resulting SRRF images had a final pixel size of 24 nm representing a 4× oversampling of the estimated spatial resolution (< 100 nm).

Image quality was evaluated by measuring the noise (variance) within cell wall regions of interest in the first acquired image of each time series, and by measuring the intensity decrease across the time series. SRRF images were examined visually for noise, granularity, patterns, or other features that might represent introduced artefacts. To quantify resolution changes resulting from SRRF processing, time series were divided into two new sequences by separating odd and even images. NanoJ Squirrel in Fiji was used to perform FRC analysis to measure the average image resolution on SRRF images generated from the odd and even sequences [57]. The same procedure was applied to average projections also generated from odd and even sequences. To further assess the effect of noise, odd and even sequences were subjected to noise reduction using PureDenoise in Fiji before SRRF processing [58].

To assess improvement in signal to noise resulting from the SRRF processing, brightness line profiles were acquired across the double cell wall from lumen to lumen at matched locations on SRRF and average projection images of the same field of view.

To compare cell wall layer dimensions in SRRF and average projection images in both wet and dry states, 10 replicate measurements were performed using several fields of view for autofluorescence, acriflavin or rhodamine B treated sections. The thickness of the compound middle lamella on tangential cell walls at matched locations was determined for both average intensity projections and SRRF (TRPPM) images using a measurement tool to apply the FWHM criterion using Digital Optics V++ software. Average intensity projections were interpolated to the same image size as SRRF images (5120 × 5120 pixels) using bilinear interpolation to allow precise positioning of each line profile in the matching projection and SRRF images. In this case, the line profile was collected between the middle of the S2 layers in adjacent cells. To provide control measurements in the dry state, comparable transverse sections were prepared for measurement by scanning electron microscopy. These measurements were performed by direct cursor measurement using Digital Optics V++ software. Measurements on projections and SRRF images were statistically analysed using a paired comparisons analysis of variance after log-transformation of data using Microsoft Excel.

### Xylem samples for polarised light microscopy

Small blocks of latewood from ring 28 were embedded in LR White acrylic resin and thin sectioned at a thickness of 1 μm using glass knives and an ultramicrotome (Leica Ultracut UCT). Sections mounted in immersion oil were examined using a Leica DM5000 polarised light microscope.

## Data Availability

Contact the author.

## Abbreviations

AFM: Atomic force microscopy
CLSM: Confocal laser scanning microscopy
CML: Compound middle lamella
FRC: Fourier ring correlation
FWHM: Full width at half maximum height
GFP: Green fluorescent protein
sCMOS: Scientific complementary metal-oxide semiconductor
SEM: Scanning electron microscopy
SRRF: Super-resolution radial fluctuations
STED: Stimulated emission depletion
TEM: Transmission electron microscopy
TRA: Temporal radiality average
TRAC: Temporal radiality autocorrelation
TRM: Temporal radiality maximum
TRPPM: Temporal radiality pairwise product mean
